# Utility of Circulating Cell-Free RNA Analysis for the Characterization of Global Transcriptome Profiles of Multiple Myeloma Patients

**DOI:** 10.3390/cancers11060887

**Published:** 2019-06-25

**Authors:** Maoshan Chen, Sridurga Mithraprabhu, Malarmathy Ramachandran, Kawa Choi, Tiffany Khong, Andrew Spencer

**Affiliations:** 1 Myeloma Research Group, Australian Centre for Blood Diseases (ACBD), Clinical Central School, Monash University, Melbourne 3004, Australia; maoshan.chen@monash.edu (M.C.); durga.mithraprabhu@monash.edu (S.M.); malarmathy.ramachandran@monash.edu (M.R.); kawa.choi@monash.edu (K.C.); tiffany.khong@monash.edu (T.K.); 2 Malignant Haematology and Stem Cell Transplantation, Alfred Hospital, Melbourne 3004, Australia

**Keywords:** myeloma, extracellular RNA, circulating transcriptome, relapse, liquid biopsy, biomarker, non-coding RNA

## Abstract

In this study, we evaluated the utility of extracellular RNA (exRNA) derived from the plasma of multiple myeloma (MM) patients for whole transcriptome characterization. exRNA from 10 healthy controls (HC), five newly diagnosed (NDMM), and 12 relapsed and refractory (RRMM) MM patients were analyzed and compared. We showed that ~45% of the exRNA genes were protein-coding genes and ~85% of the identified genes were covered >70%. Compared to HC, we identified 632 differentially expressed genes (DEGs) in MM patients, of which 26 were common to NDMM and RRMM. We further identified 54 and 191 genes specific to NDMM and RRMM, respectively, and these included potential biomarkers such as LINC00863, MIR6754, CHRNE, ITPKA, and RGS18 in NDMM, and LINC00462, PPBP, RPL5, IER3, and MIR425 in RRMM, that were subsequently validated using droplet digital PCR. Moreover, single nucleotide polymorphisms and small indels were identified in the exRNA, including mucin family genes that demonstrated different rates of mutations between NDMM and RRMM. This is the first whole transcriptome study of exRNA in hematological malignancy and has provided the basis for the utilization of exRNA to enhance our understanding of the MM biology and to identify potential biomarkers relevant to the diagnosis and prognosis of MM patients.

## 1. Introduction

Multiple myeloma (MM) is a neoplasm of terminally differentiated plasma cells (PC), which at diagnosis is usually present throughout the bone marrow (BM) and frequently, with disease progression, spreads to extramedullary locations. The treatment of MM has witnessed significant progress with the introduction of proteasome inhibitors and immunomodulatory agents; however, the disease remains incurable with MM cells acquiring resistance to systemic therapies due to the accumulation of additional mutations that are often not present during the initial stages of the disease [1]. Resistance to therapy is mediated through the genetic evolution of MM cells under selective pressure treatment with more resistant clones emerging that possess growth and survival advantages [2].

Next-generation deep sequencing of MM tumor specimens has identified widespread genetic heterogeneity in MM [3,4]. Gene expression profiling (GEP) studies of PC isolated from patients presenting with the pre-MM precursor syndrome, monoclonal gammopathy of undetermined significance (MGUS), and symptomatic MM, have identified specific transcriptional changes that occur at different stages of the disease and these studies have reported pathways that had not been previously implicated in MM pathogenesis [5]. The current practice for obtaining genetic information about the tumor is to perform sequential BM biopsies and this is confounded by the known inter and intra-clonal spatial and genetic heterogeneity of the tumor(s) [1,2,6]. An alternative approach that we believe will provide a more comprehensive transcriptional picture is to analyze the extracellular RNA (exRNA) from the peripheral blood (PB) plasma, as this theoretically contains genetic material that arises not only from multiple independent MM tumors but also from the tumor microenvironment (TME).

Nucleic acids are released into the plasma and serum through cellular apoptosis, necrosis, and the spontaneous release of DNA/RNA-lipoprotein complexes, amongst other sources [7]. The presence of cell-free (cf) nucleic acids in the human blood was described more than 60 years ago [8]. A number of recent studies in breast, ovarian, lung, and colorectal cancers have indicated that tumor(s) from both primary and secondary sites release nucleic acids into the PB, as such, circulating cfDNA contains a representation of the entire genome of the tumor with DNA sourced from multiple independent tumors. Whole genome sequencing and whole exome sequencing of the cfDNA can be utilized to identify mutations associated with acquired resistance to cancer therapy without the need to perform sequential biopsies of the tumor [9,10]. Also, some studies have uncovered the presence of microRNAs and specific long noncoding RNAs in the exosomes, an extracellular vesicle subtype ranging from 30–150 nm in size [11], isolated from the blood of MM patients [12,13]. However, exRNA has not been systematically explored, with only a limited number of studies exploring the relevance of specific mRNA transcripts [14,15,16,17,18]. More recently, technologies have advanced that promote the reliable extraction and analysis of exRNA using high-throughput sequencing technologies, and a limited number of studies have also performed whole transcriptome studies of exRNA in pregnancy and Alzheimer’s disease [19,20,21]. However, to our knowledge, no studies have explored the whole transcriptome of exRNA in MM or other cancers. 

Global gene expression profiling of PC from normal, MGUS, smoldering (asymptomatic) myeloma (SMM), and symptomatic MM patients has indicated that MGUS patients have a profile that is different to SMM and MM patients and that a gene-based classification system can be employed for MM [22,23,24]. In this study, we profiled the whole transcriptome of exRNA obtained from the PB plasma of healthy controls (HC) and MM patients to identify potential biomarkers for both newly diagnosed (NDMM) and relapsed and refractory (RRMM) MM patients.

## 2. Materials and Methods

### 2.1. Peripheral Blood (PB) Collection and Processing

Peripheral blood (PB) samples were obtained from healthy volunteers (HC) and MM patients using 10 mL Streck RNA blood collection tubes (BCTs) following informed consent as per the Alfred Hospital Human Research and Ethics Committee. Immediately upon sample collection, the tubes were inverted to mix the blood with the preservative in the collection tube. The patented preservative in Cell-Free RNA BCT preserves exRNA in plasma and prevents the release of non-target background RNA from blood cells during sample processing and storage [25,26]. The Streck RNA tubes allow the collection of high-quality stabilized exRNA for rare target detection and determining accurate exRNA concentrations. Streck RNA BCT tubes were stored at room temperature and processed for plasma collection within 24 h of PB collection. Plasma was separated from PB through centrifugation at 820× *g* for 10 min. The supernatant was collected without disturbing the cellular layer and centrifuged again at 16,000× *g* for 10 min to remove any residual cellular debris. The supernatant was collected and stored at −80 °C until exRNA extraction.

### 2.2. exRNA Extraction

Frozen plasma samples (6 mL) were used to extract the exRNA using the QIAamp circulating nucleic acid kit (Qiagen, Hilden, Germany), according to the manufacturers’ instructions. Subsequently, a Qubit 2.0 Fluorometer (Life Technologies, Victoria, Australia) was used to measure the RNA yield, according to the manufacturer’s instructions with freshly prepared RNA standards. The maximum input volume utilized for the QUBIT assay was 5 μL. The RNA concentration was quantified as being between 0.5–90 ng/μL. The extracted RNA was stored at −80 °C until further processing for RNA-Seq.

### 2.3. Transcriptome Library Construction and Sequencing 

After the RNA sample was treated using the Ribo-Zero Gold rRNA Removal Kit (Illumina, San Diego, CA, USA), a total amount of 100 ng RNA was used for cDNA library construction using the TruSeq™ RNA Library Preparation Kit v2 protocol (Illumina, San Diego, CA, USA), as previously described [27]. Then, the amplified cDNA libraries were assessed for quality using both the Agilent 2100 Bioanalyzer and qRT-PCR. After primer ligation sequencing, qRT-PCR was again used for quantification, and the final library was sequenced on an Illumina HiSeq 2000 platform (paired-end 100 bp) at the Australian Genomics Research Facility (Melbourne, Australia).

### 2.4. Analysis of Transcriptome Data

Raw transcriptome sequencing data were cleaned using SOAPnuke under default parameters [28]. The clean reads were aligned to the human reference genome (ENSEMBL, GRCH38.85) using Hisat2 and the expression of protein-coding and noncoding genes were profiled using Stringtie [29]. HTSeq (v0.9.0, https://htseq.readthedocs.io) was used to obtain the raw read counts aligned to each gene. 

The TPM (transcripts per million reads) method was used to normalize the gene expression in each sample, and lowly expressed (<5 TPM) genes were filtered. To perform differential expression analysis, we first removed individual differences among samples by filtering genes with high coefficient values (>0.8) of standard variation in each group. An R package named as edgeR was used to calculate the statistical values for differentially expressed genes (DEGs) [30] and the DEGs were identified based on the following criteria: TPM > 5, log2 fold change (Log2FC) > 1 or Log2FC < −1, *p*-value < 0.05 and FDR < 0.05. Principle component analysis was performed using the R package “prcomp”. Genome alignment files could be accessed on the National Center for Biotechnology Information (NCBI) platform under the accession number SRA893057.

### 2.5. Functional Analysis

Functional analysis of DEGs was performed using the DAVID Bioinformatics Resources 6.8 (https://david.ncifcrf.gov).

### 2.6. Identification of SNP and Indel Variations

Variants including SNPs and indels were called using the Genome Analysis Toolkit (GATK v3.4) pipeline, according to the Best Practice workflow. In brief, the clean reads for each sample were aligned to the human reference genome (GRCH38) using the STAR 2-pass alignment steps [31]. Then, the resulting SAM file was put through the usual Picard processing steps, including adding read group information, sorting, marking duplicates, and indexing (http://broadinstitute.github.io/picard/). Next, the RNA-Seq specific GATK tool called SplitNCigarReads was used to split the reads into exon segments and hard-clip any sequences overhanging into the intronic regions. After the indel realignment and base recalibration, we used HaplotypeCaller to call SNPs and indels, followed by variant filtering with recommended commands and parameters. We further filtered the results by removing the variants detected in less than half of the sample size. Final variants in MM patients excluded the events detected in HC. ANNOVAR was used to annotate the variants [32].

### 2.7. Droplet Digital PCR

We randomly selected 10 samples used for RNA-Seq, including three HC, two NDMM, and five RRMM patients for gene expression validation using droplet digital PCR (ddPCR). Initially, 3 mL of plasma samples were used for exRNA extraction, as described. Then, a NanoDrop 1000 was used to quantify the RNA yield. One-Step RT-ddPCR Advanced Kit for Probes (Bio-Rad Laboratories, Hercules, CA, USA) was used for the detection of five DEGs, including RGS18 (dHsaCPE5054808), ITPKA (dHsaCPE5192800), PPBP (dHsaCPE5039499), FTH1 (dHsaCPE5031151), and IER3 (dHsaCPE5190327), according to the manufacturer’s instructions. Briefly, the reaction mix (22 µL) was prepared with 5 µL Supermix (1×), 2 µL reverse transcriptase (20 U/µL), 1 µL DTT (300 nM), 1 µL gene probe (900 nM), 1 µL total RNA, and 12 µL RNase-free water. Then, the droplets were generated on the Automated Droplet Generator (Bio-Rad), followed by the thermal cycling reaction. The cycling conditions were conducted on a C1000 Touch Thermal Cycler (Bio-Rad) as follows: reverse transcription (46 °C, 60 min), enzyme activation (95 °C, 10 min), 40 cycles of denaturation (95 °C, 30 s), and annealing/extension (60 °C, 1 min), and enzyme deactivation (98 °C, 10 min). After thermal cycling, the plates were read by the QX200 Droplet Reader (Bio-Rad) and the QuantaSoft software was used to analyze the data. To reduce the errors, we employed GAPDH (Glyceraldehyde-3-Phosphate Dehydrogenase) as an internal control and the gene expression was shown relative to the absolute copies of GAPDH. Aberrant values (>500 copies/µL) were set to null, and samples with no gene expression were not counted for the calculation of the average gene expression.

## 3. Results

### 3.1. Characteristics of MM Patients and RNA Profiles

To investigate the biomarker potential of exRNA for NDMM and RRMM, this study recruited a total of 27 volunteers, including 10 HC (six males and four females), five NDMM (three males and two females) patients, and 12 RRMM (seven males and five females) patients. No difference was found between the groups based on gender, moreover, as shown in Table 1, the mean age of NDMM (66 years) and RRMM (64.6 years) were also similar (older than the HC group: 41.9 years). After the total RNA was extracted, we evaluated the RNA profiles of exRNA using an Agilent Bioanalyzer 2100. Unlike the RNA profiles of whole cell lysates, the exRNA profiles lacked the 18S and 28S rRNA peaks (Supplementary Figure S1). Whether this is one of the characteristics of exRNA requires further experiments to explore because in this instance, the exRNA sample was treated with the Ribo-Zero Gold rRNA Removal Kit.

### 3.2. Overview of the Transcriptome Sequencing

**Figure 1:**
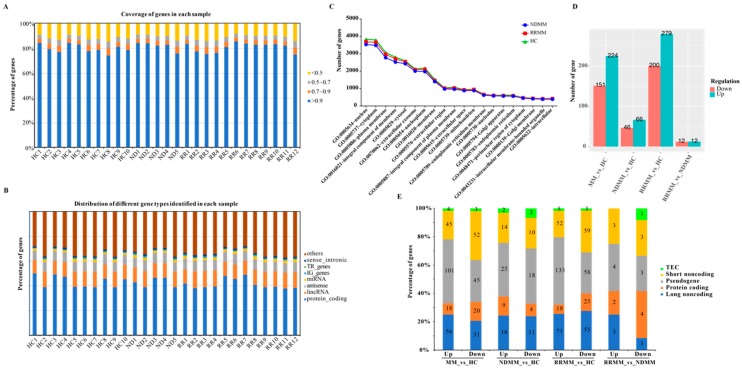
Overview of the transcriptome of extracellular RNA (exRNA) in healthy controls (HC) and multiple myeloma (MM) patients. (**A**) Distribution of gene coverage in each sample. (**B**) Distribution of gene types in each sample. (**C**) Top 20 terms of the GO cellular component identified by the genes in HC, newly diagnosed multiple myeloma (NDMM), and relapsed and refractory multiple myeloma (RRMM). (**D**) Status of differentially expressed genes identified in MM compared to HC. (**E**) Distribution of gene types for the differentially expressed genes (DEGs).

After low-quality reads, adapter and contamination reads were filtered by SOAPnuke; we obtained a total of 762.50 million paired reads (28.24 million reads per sample on average) (Table 2). The lowest Q20 and Q30 of this sequencing were 93.83% and 86.53%, respectively (Table 2). Using Hisat2, 88.40–96.78% of the clean reads were aligned to the human reference genome (GRCH38), and 83.20–94.73% of the clean reads were paired matched (Table 2). Then, we used Stringtie to profile the gene expression in each sample. As shown in Table 2, 23,274 to 38,293 genes were identified in the MM patients and HC, respectively. We next evaluated the coverage of identified genes in each sample and found that 79.76–88.58% of the identified genes were covered more than 70% by the sequencing reads (Figure 1A). Gene annotation showed that 38.71–50.51% of the identified genes had the potential of protein-coding capacity while 1.77–2.33% of the identified genes were to be experimentally confirmed (TEC) (Figure 1B). Three long-noncoding RNAs, including RN7SL1, RN7SL2, and RN7SL4P, were the most highly expressed genes in the HC, NDMM, and RRMM samples, followed by the Metazoa_SRP (Metazoan signal recognition particle RNA).

We next analyzed the gene ontology in terms of the cellular component to source the exRNA molecules in cells. The top 20 enriched cellular components of GO were shared by NDMM, RRMM, and HC (Figure 1C). With the exception of the nucleus and the cytoplasm, the genes were enriched in the membrane and extracellular vesicle-related GO terms, such as the “plasma membrane (GO:0005886)” and “extracellular exosome (GO:0070062)”.

### 3.3. Dysregulated exRNA in MM Patients

To identify potential exRNA biomarkers for NDMM and RRMM, we performed differential gene expression analysis using edgeR under the criteria described in the methods. First, we compared all the MM (NDMM + RRMM) patients to the HC and identified 375 DEGs (224 up-regulated and 151 down-regulated) (Figure 1D, Supplementary Table S1). As shown in Figure 1E, 38 protein-coding genes (18 up-regulated and 20 down-regulated), 87 long noncoding genes (56 up-regulated and 31 down-regulated), 97 short noncoding genes (45 up-regulated and 52 down-regulated), and seven TEC genes (four up-regulated and three down-regulated) were differentially expressed in MM compared to HC. Apart from the immunoglobulin-related genes, GOLGA8O (golgin A8 family member O) and KCTD14 (potassium channel tetramerization domain containing 14) were the top two up-regulated genes, while the top two down-regulated genes were C21orf33 (chromosome 21 open reading frame 33) and RPL13A (ribosomal protein L13a).

Next, compared to HC, we identified 66 up-regulated and 46 down-regulated genes (Figure 1D, Supplementary Table S1) in NDMM, including 13 protein-coding genes (nine up-regulated and four down-regulated), 27 long noncoding genes (16 up-regulated and 11 down-regulated), 43 pseudogenes (25 up-regulated and 18 down-regulated), 24 short noncoding genes (14 up-regulated and 10 down-regulated), and five TEC (two up-regulated and three down-regulated) (Figure 1E). The nine up-regulated protein-coding genes were MYOD1, AC009060.2, CHRNE, GOLGA8O, NKX2-4, FTH1, UBB, OLIG1, and ITPKA. The four down-regulated protein-coding genes were RGS18, HISTH1B, TRK2, and MBD3L5. 

In RRMM, we identified 279 up-regulated and 200 down-regulated genes compared to HC (Figure 1D, Supplementary Table S1), including 43 protein-coding genes (18 up-regulated and 25 down-regulated), 126 long noncoding genes (71 up-regulated and 55 down-regulated), 191 pseudogenes (133 up-regulated and 58 down-regulated), 111 short noncoding genes (52 up-regulated and 59 down-regulated), and eight TEC (five up-regulated and three down-regulated) (Figure 1E). The highly up-regulated protein-coding genes included immunoglobulin genes and GOLGA8O, while the highly down-regulated protein-coding genes included HIST1H4A and RPL13A.

### 3.4. Potential exRNA Biomarkers for MM Patients

**Figure 2:**
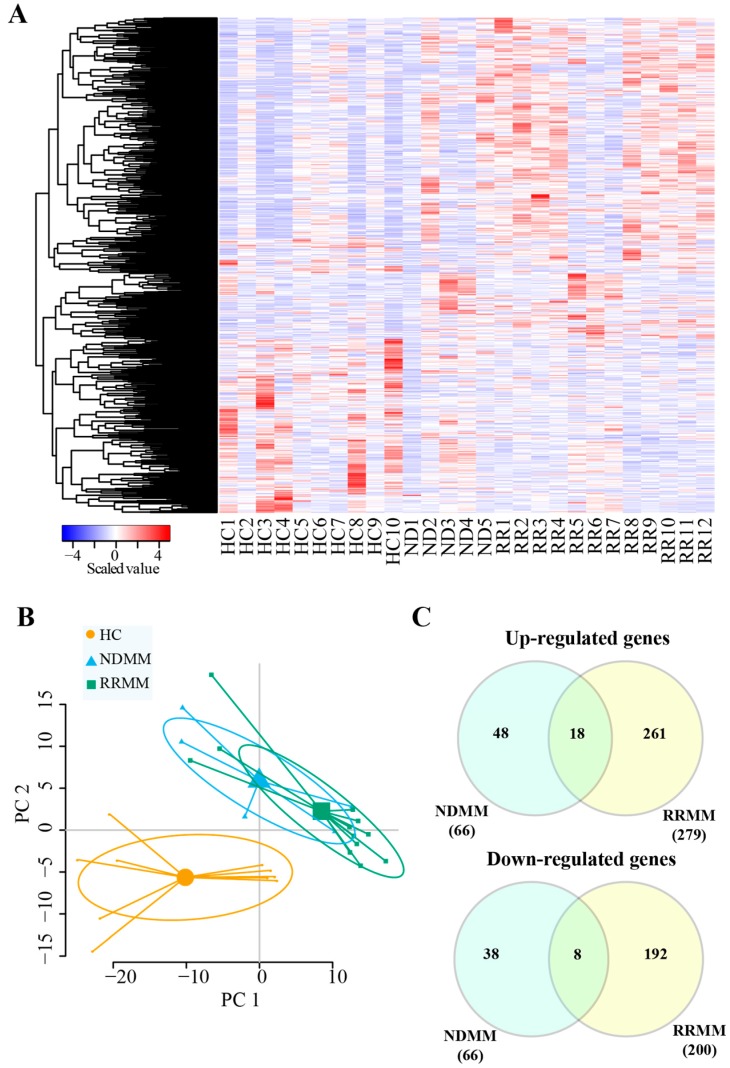
Comparison of DEGs in NDMM and RRMM. (**A**) A heat map of the DEGs showed different signatures for HC, NDMM, and RRMM. (**B**) Principle component analysis of all samples. (**C**) Venn diagrams of up- and down-regulated genes identified in all MM patients, NDMM, and RRMM.

A heat map (Figure 2A) showed that DEGs could be distinguished between MM patients and HC. This was also revealed by the principal component analysis (Figure 2B). However, due the limited sample size of the NDMM, it was difficult to separate NDMM from RRMM (Figure 2B). We next compared the DEGs identified in NDMM and RRMM. As shown in Figure 2C, 18 up-regulated and eight down-regulated genes were common to MM (NDMM + RRMM), including one up-regulated protein-coding gene (GOLGA8O) and one down-regulated protein-coding gene (TRAK2). We also identified 48 up-regulated and 38 down-regulated genes specific to NDMM (Figure 2C, Supplementary Table S1), which could be utilized as potential biomarkers for NDMM. The NDMM specific up-regulated genes included seven lincRNA genes (e.g., AC007405.6, LINC00863, RP11-573G6.10, and RP11-626H12.1), one primary miRNA gene (MIR6754), and eight protein-coding genes (e.g., MYOD1, AC009060.2, CHRNE, NKX2-4, FTH1, UBB, OLIG1, and ITPKA). The NDMM specific down-regulated genes included five lincRNA genes (e.g., PACERR, LINC01123, RP11-596C23.2, and AC137934.1) and three protein-coding genes (e.g., MBD3L5, RGS18 and HIST1H1B). In addition, we identified 261 up-regulated and 192 down-regulated genes specific to RRMM (Figure 2C, Supplementary Table S1). The RRMM specific dysregulated exRNA genes included 34 protein-coding genes (11 up-regulated and 23 down-regulated), 52 lincRNA genes (33 up-regulated and 19 down-regulated), 18 primary miRNA genes (10 up-regulated and eight down-regulated), and 37 misc_RNA genes (18 up-regulated and 19 down-regulated). 

Next, we compared the exRNA profiles of NDMM and RRMM. Using edgeR we identified a total of 24 genes differentially expressed in RRMM relative to NDMM, including 12 up-regulated and 12 down-regulated genes (Figure 1D). The distribution of different gene types in the DEGs of RR and ND (Figure 1E) showed that six protein-coding DEGs, including two up-regulated genes (IGKV1-5 and OR4N4) and four down-regulated genes (AC017081.1, UBB, CHRNE, and USP17L17) were dysregulated in RRMM compared to NDMM. Among them, UBB and CHRNE were up-regulated in NDMM and UBB was also up-regulated in MM (NDMM + RRMM) (Supplementary Table S1). Also, we found a lincRNA gene AC137934.1 up-regulated in RRMM compared to NDMM but down-regulated in NDMM compared to HC. 

Functional analysis using DAVID bioinformatics resources showed that 10 and 11 DEGs in RRMM compared to HC were involved in the biological process of “SRP-dependent co-translational protein targeting to membrane” (GO:0006614) and “Ribosome” (hsa03010) pathways, respectively. Also, two DEGs in NDMM compared to HC were involved in the molecular function of “chromatin DNA binding” (GO:0031490). According to the Kyoto Encyclopedia of Genes and Genomes (KEGG) pathway annotation, we also found that ITPKA, which was up-regulated in NDMM, was related to signal transduction. Among the DEGs in the RRMM, PPBP (down-regulated) and CCL4L2 (up-regulated) were involved in the “chemokine signaling pathway” (ko04062), HIST1H4A (down-regulated) was related to “Viral carcinogenesis” (ko05203), and PCBP1 (down-regulated) was a regulator of “spliceosome” (ko03040). 

### 3.5. Gene Variants in MM Patients

**Figure 3:**
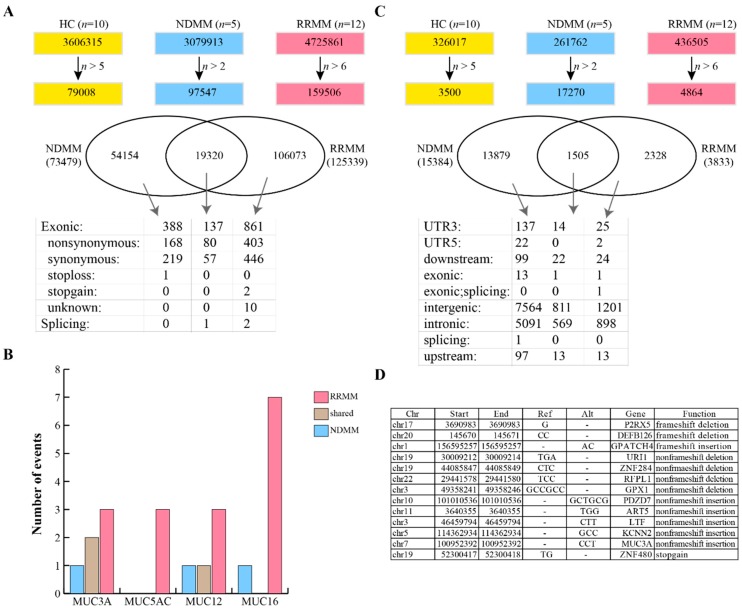
SNP and indel analysis. (**A**) Identification of SNPs in MM and their annotation. (**B**) Comparison of the distribution and the frequency of mucin gene mutations in NDMM and RRMM. (**C**) Identification of small indels in NDMM and RRMM. (**D**) Annotation of 13 known small indels specific to NDMM.

We next used the GATK pipeline to detect SNPs and small indels in MM patients. As shown in Figure 3A, we first identified 3,606,315 SNPs in HC, of which 79,008 were found in more than five individuals. We also identified 97,547 SNPs in more than two NDMM and 159,506 SNPs in more than six RRMM. The SNPs identified in HC were classified as background and were filtered from the MM patients, resulting in 734,79 and 125,399 SNPs in NDMM and RRMM, respectively. There were 19,320 SNPs shared between NDMM and RRMM (Figure 3A). ANNOVAR was used to annotate the SNPs, and the results showed that 137 exonic SNPs were identified in both NDMM and RRMM, including one splicing event, 80 nonsynonymous, and 57 synonymous single-nucleotide variants (SNVs) (Figure 3A). The shared nonsynonymous SNPs all have been annotated in dbSNP (Table 3). Seven SNPs were identified in more than four NDMM patients and more than 10 RRMM patients, including rs61821060, rs2363468, rs1941635, rs2172521, rs7199961, rs73714227, and rs4728137. rs61821060 is a mutation of the RPFIA4 gene (chr1 203039046, G->C) and was found in five NDMM and 11 RRMM patients. There were 388 and 861 exonic SNPs specific to NDMM and RRMM, respectively (Figure 3A). The 388 NDMM specific exonic SNPs included 168 nonsynonymous (Supplementary Table S2), 219 synonymous, and one stop-loss SNP. Notably, we found one nonsynonymous SNP (chr7, 100958135, T->C, MUC3A) that has not been reported in the Single Nucleotide Polymorphism Database (dbSNP). The 861 RRMM specific exonic SNPs included 403 nonsynonymous (Supplementary Table S3), 446 synonymous, two stop-gain and 10 unknown SNPs. Of the 403 nonsynonymous SNPs we identified five novel mutations, two occurred in MUC5AC (chr11, 1191524, T->C; chr11, 1191527, C->T), and three in MUC3A (chr7, 100953992, C->A; chr7, 100954123, G->A; chr7, 100954136, C->G). Next, we analyzed the mutations occurring in intestinal mucin genes, such as MUC3A, MUC5AC, MUC12 and MUC16. It was revealed that more SNPs occurred in mucin genes in RRMM than in NDMM (Figure 3B), particularly the MUC5AC gene.

We next evaluated for the presence of indels in the MM patients. As shown in Figure 3C, a total of 15,384 and 3833 indels were identified in NDMM and RRMM, respectively. NDMM and RRMM shared 1505 indels, of which one was derived from the exonic region. The shared exonic indel was annotated as rs11402251, which is located in the 10th exon of the VSIG10L gene and is a frameshift insertion. Two known indels (rs5843453 and rs55745992) were identified specifically in RRMM, while 13 known indels were identified specifically in NDMM (Figure 3D). Interestingly, nine out of the 13 NDMM specific indels had no frameshift function to their mother genes. The left indels included frameshift deletions on P2RX5 and DEFB126, frameshift insertions to GPATCH4, and a stop-gain insertion to ZNF480 (Figure 3D).

Functional analysis by DAVID showed that mutated genes for both NDMM and RRMM were involved in “Olfactory transduction (hsa04740)” and “GO:0007186~G-protein-coupled receptor signaling pathway”. In addition, RRMM specific mutated genes were also involved in “ECM-receptor interaction (hsa04512)”, “GO:0007155~cell adhesion”, “GO:0071813~lipoprotein particle binding”, and “PI3K-AKT signaling pathway (hsa04151)”.

### 3.6. Digital Droplet PCR Validation

**Figure 4:**
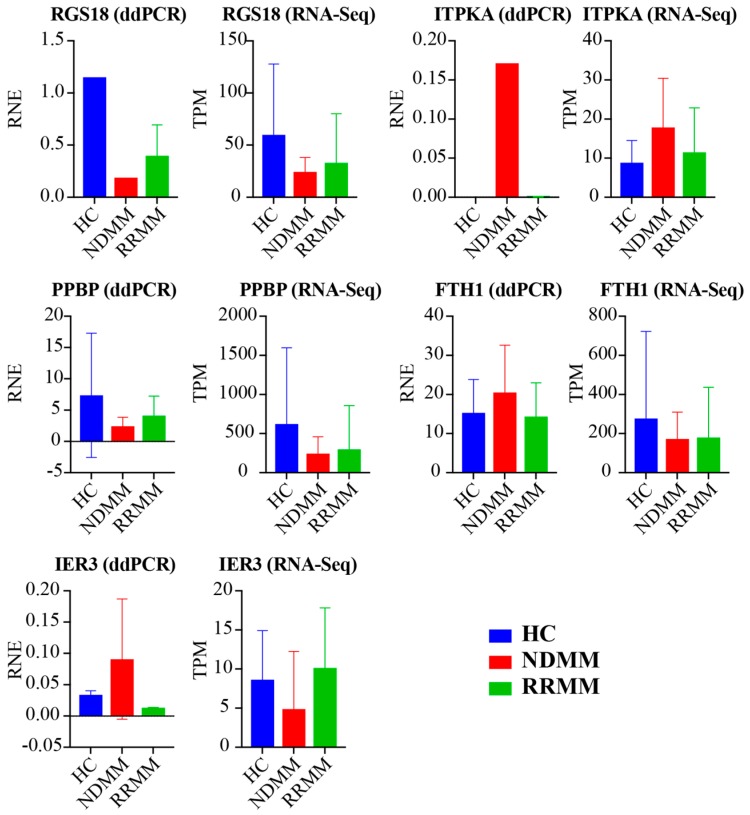
Digital Droplet PCR (ddPCR) validation. Five DGEs were selected and compared to RNA-Seq. Their expression was normalized to GAPDH and was present in relative normalized expression (RNE) by ddPCR. Their normalized expression identified by RNA-Seq was shown in transcripts per million reads (TPM).

We next utilized ddPCR to validate five DEGs (RGS18, ITPKA, PPBP, FTH1, and IER3) in the exRNA samples from 10 participants (three HC, two NDMM, and five RRMM). We used relative normalized expression (RNE, relative to GAPDH) to present the gene expression detected by ddPCR. Figure 4 showed the comparison of gene expression identified by ddPCR and RNA-Seq. Among them, the expression levels of three genes (RGS18, ITPKA, and PPBP) were consistent between these two experiments in HC, NDMM, and RRMM. In addition, FTH1 was up-regulated in NDMM compared to HC (Log2FC = 1.78, FDR = 2.16 × 10^−7^, Supplementary Table S1) and its up-regulation was also confirmed by ddPCR. The abnormal performance of FTH1 in Figure 4 is due to its high expression in the three HCs. The disagreement of ddPCR and RNA-Seq on the expression change of IER3 require further experiments for validation. 

## 4. Discussion

In this study, we evaluated plasma-derived exRNA from MM patients and healthy individuals (Table 2). The exRNA has been reported to be subjected to degradation, instability, low abundance, and intracellular communication from specimen processing [33,34]; however, in our study we demonstrated that 79.76–88.58% of the identified genes were covered more than 70%, which means that a large set of gene transcripts was complete in the exRNA tested. The stability of exRNA in the plasma or serum may be due to the protection of extracellular vesicles, such as exosomes, microparticles, microvesicles, or multivesicles [35], which are shed from cellular surfaces into the bloodstream [11]. In support of this, we observed that the RNA profiles generated by the Agilent Bioanalyzer 2100 were similar to exosomal RNA profiles [36], which lack 18S and 28S rRNA peaks. Furthermore, our results showed that 2002–2135 genes were related to the “extracellular exosome (GO:0070062)” (Figure 1C). New evidence has shown that a large proportion of human blood plasma cf-DNA is localized in exosomes [37]; however, the proportion of exosomal RNA in exRNA requires further investigation. 

No studies have investigated the global transcriptome profiles of exRNA in MM. In this study, we identified genes in exRNA that could potentially be used as diagnostic and prognostic biomarkers for MM, including 18 up-regulated and eight down-regulated genes (Figure 2B, Supplementary Table S1). GOLGA8O was the only up-regulated protein-coding gene common to both NDMM and RRMM. It has been reported to be significantly down-regulated in patients with major depressive disorder [38]; however, the function of this gene in cancers has not been investigated. The common down-regulated genes in MM included TRAK2, a possible regulator of endosome-to-lysosome trafficking of membrane cargo, including the epidermal growth factor receptors (EGFR) [39]. Endosome-to-lysosome trafficking is also an important process in exosome biogenesis [11]. In addition, TRAK1, the ortholog of TRAK2, has been identified as MGb2-Ag—a novel cancer biomarker [40]. In MM, the down-regulation of TRAK2 might be due to the up-regulation of miR-19a [41,42], which is a member of miR-17~92a cluster and can target the TRAK2 gene [41].

In this study, we identified 54 genes specifically dysregulated in NDMM, including MYOD1, UBB, MIR6754, and PACERR (Supplementary Table S1). The hypermethylation of MYOD1 has been reported to be a prognostic biomarker for both colorectal and cervical cancers [43,44]. The polyubiquitin gene UBB, encoding a regulatory protein involved in ubiquitin, has been identified to be up-regulated more than 100-fold in MM patient cells versus normal twin plasma cells [45]. Moreover, the knockdown of UBB can significantly decrease the expression of ubiquitin, which is essential for cancer cell growth. UBB may, therefore, represent a potential target in anticancer treatment including in MM [46]. miR-6754 has been related to cell proliferation and invasion in non-small cell lung cancer [47]. PACERR is the antisense gene of PGTS2 (prostaglandin-endoperoxide synthase 2) that has been related to colorectal cancer and breast cancer [48,49]. There are currently no published studies that relate MIR6754 and PACERR or other noncoding RNAs to MM; however, their expression patterns in NDMM suggest that they might play a role in the progression of MM and may also represent potential biomarkers for MM.

Among the 191 genes specifically dysregulated in RRMM patients, seven down-regulated (PPBP, EEF1B2, RPL5, RPL19, CH507-9B2.3, EEF2, and RPS27) and three up-regulated (VN1R1, IER3 and POTED) protein-coding genes were identified (Supplementary Table S1). Studies have reported the overexpression of most of these genes in multiple cancers, with the exception of CH507-9B2.3 [50,51,52,53,54]; however, they have also been found to be down-regulated in some specific cancers. For example, the chemokine PPBP (pro-platelet basic protein), also known as CXCL7, is decreased in pancreatic and ovarian cancers [55]. The eukaryotic translation elongation factors EEF1B2 and EEF2 are down-regulated in breast, esophageal, and lung cancers while EEF1B2 is found with repression in some other cancer types, such as head and neck, leukemia, and pancreatic cancer [54]. Whether the down-regulation of these genes is specific to MM requires further study. Only three protein-coding genes were specifically up-regulated in RRMM, including VN1R1, POTED, and IER3 (Supplementary Table S1). Among them, VN1R1 has been shown to be overexpressed in prostate adenocarcinomas and glioblastoma cancer cells [56,57] and POTED in prostate cancer patients, making it a potential molecule for targeted therapy [58]. Interestingly, IER3 has been shown in pancreatic ductal adenocarcinoma to effectively mitigate against cellular stress induced by starvation or exposure to gemcitabine [59]. Notably, IER3 has also been demonstrated to have an anti-apoptotic role in MM endothelial cells and is overexpressed in MM plasma cells [60].

In comparison to protein-coding genes, a larger proportion of noncoding DEGs were up-regulated in MM (Supplementary Table S1), which is consistent with a previous study, albeit of PC-derived RNA [61]. Some lncRNAs are considered to be potential promoters of MM progression, and thus, could have potential as diagnostic and prognostic biomarkers, these include LINC01215 [62], MIR222HG [62], MEG3 [63], MALAT [64], CRNDE [65], and H19 [66]. In this study, additional lncRNAs with biomarker potential in MM were identified (Supplementary Table S1), including 65 antisense genes (e.g., FAM83C-AS1, ZNF32-AS1, TMC3-AS1, and TAT-AS1), 71 lincRNA genes (e.g., LINC00863, LINC01123, LINC00349, LINC00677, and LINC00462) and 25 primary miRNA genes (e.g., MIR301A, MIR378H, MIR425, and MIR647). Among these, some have been reported in other cancers, such as LINC01123 in prostate cancer [67], LINC00677 in acute myeloid leukemia [68] and LINC00462 in pancreatic cancer [69]. The function of these lncRNAs in MM and their diagnostic and prognostic potential requires further evaluation.

In this study, we also identified gene variants using the transcriptome data (Figure 3). Of interest were the RRMM specific indels related to both cell adhesion and PI3K-AKT signaling pathways. Unfortunately, the hotspot mutations on KRAS, NRAS, and TP53 genes, which are common in MM [70], were not identified in this study, probably due to the low coverage of these genes [71]. However, our findings revealed that mucin genes, such as MUC3A, MUC5AC, MUC12, and MUC16, may play a role in MM progression, as they were frequently mutated in the MM patient, particularly the RRMM patients. Moreover, MUC16 mutations, the most frequently mutated in this analysis, have been previously demonstrated to be associated with a higher tumor mutational burden and superior survival outcomes in gastric cancer patients [72]. Additionally, uterine endometrioid endometrial adenocarcinoma harbor a high-frequency of the MUC3A mutations [73]. Whether mucin gene variants are derived from MM is still unknown, and our results indicate that mucin genes might be related to the MM pathogenesis. These results demonstrate the potential capacity of exRNA interrogation to identify genetic variants; however, we acknowledge that more sequencing data (high-coverage) and DNA sequencing are required to validate the genomic mutations of the described mucin genes and more patient samples are needed for large scale scanning.

## 5. Conclusions

In conclusion, this is the first transcriptome study of exRNA in cancer and in our hands, we have demonstrated that ~85% of genes in exRNA were covered more than 70% and that ~45% of exRNA genes were protein-coding genes. DEGs identified in MM patients, including GOLGA8O and TRAK2 may be potential biomarkers for the disease. Importantly, we also identified specific differentially expressed protein-coding genes in both NDMM and RRMM including cancer-associated genes such as MYOD1, UBB, VN1R1, POTED, and IER3, and while they may have potential as biomarkers also warrants further study to determine their potential role in the pathogenesis of the disease. In addition, a range of nonsynonymous mutations were identified in the exRNA, including multiple mutated mucin genes and their role in MM also warrants further evaluation. While preliminary, these data demonstrate that exRNA may represent a valuable, non-invasive compartment not only for the identification of new diagnostic and prognostic biomarkers in MM but also for the study of the biology of the disease.

## Figures and Tables

**Table 1:** Description of participants in this study. No bias was found in age or sex in the participants for each group. HC: healthy control; ND: newly diagnosed MM patient; RR: relapsed and refractory MM patient; IgA: immunoglobulin A; FISH: fluorescence in situ hybridization. Translocation t(4:14) represents the FGFR3-IGH fusion event while t(14:16) represents the IGH-MAF fusion event.

Group	Sample	Age	Sex	IG_Type	Cytogenetics
Healthy control	HC1	61	F		
	HC2	36	M		
	HC3	59	M		
	HC4	65	M		
	HC5	28	F		
	HC6	64	F		
	HC7	28	M		
	HC8	28	M		
	HC9	25	F		
	HC10	25	M		
NDMM	ND1	84	M		
	ND2	75	F		
	ND3	52	M	IgA kappa	t(4;14)
	ND4	54	M	IgA kappa	FISH negative for 17p, t(4;14), t(14;16)
	ND5	65	F	IgA lambda	t(14;16)
RRMM	RR1	66	M	IgG Lambda	High risk +1q
	RR2	69	F		
	RR3	62	M	IgA kappa	t(4;14) in 94% of cells on FISH
	RR4	60	M		
	RR5	62	F		
	RR6	53	F	IgG kappa 12 g/L	
	RR7	57	M		Del 16q
	RR8	64	M	IgG lambda	
	RR9	71	F	Kappa Light chains	
	RR10	63	M	IgG lambda	
	RR11	77	F	IgG kappa	
	RR12	71	M	Lambda light chains	

NDMM—newly diagnosed multiple myeloma; RRMM—relapsed and refractory multiple myeloma.

**Table 2:** Overview of the extracellular RNA (exRNA) transcriptome sequencing. Q20 and Q30 were applied to the clean reads. The number of mapped reads was given by Hisat2 and ‘.5’ means that one of the paired reads was mapped. Percentages were calculated for mapped reads and paired reads matched to the genome. The numbers of genes identified in the samples (>5 transcripts per million reads (TPM)) were listed.

Sample	Clean Reads ^a^	Q20 (%)	Q30 (%)	Mapped Reads ^b^	Percentage (%)	Paired Mapping	Percentage (%)	Genes
HC1	35514538	94.33	87.06	32335527	91.05	30805744	86.74	25298
HC2	32851123	94.46	87.49	29510065.5	89.83	28039748	85.35	26253
HC3	35037913	94.62	87.77	31689161	90.44	30111659	85.94	26193
HC4	20540654	95.99	93.73	19878330	96.78	19459041	94.73	32587
HC5	20440423	96.36	94.31	19465740	95.23	19125751	93.57	37620
HC6	21051811	96.36	94.33	20066691	95.32	19697560	93.57	36927
HC7	20663077	96.44	94.44	19753850	95.60	19378872	93.79	37271
HC8	21280234	96.38	94.34	20188191.5	94.87	19770401	92.90	37180
HC9	20233979	96.37	94.33	19270979.5	95.24	18855816	93.19	37431
HC10	25312716	95.33	88.25	24087393.5	95.16	23479329	92.76	34429
ND1	28398995	95.66	89.04	26841732.5	94.52	26201952	92.26	35206
ND2	30255712	94.45	86.67	28480977	94.13	27722552	91.63	28239
ND3	30676993	95.38	88.42	28961701	94.41	28258901	92.12	33733
ND4	32996329	95.44	88.45	31421035.5	95.23	30717044	93.09	33671
ND5	29687796	95.15	88.03	27781572.5	93.58	27023016	91.02	34465
RR1	23654835	96.18	94.07	22562264	95.38	22117174	93.50	38293
RR2	34826086	95.48	89.72	31683769.5	90.98	29855275	85.73	24750
RR3	34519866	95.27	89.64	31195727	90.37	29771168	86.24	27030
RR4	19760808	96.19	94.09	18778665.5	95.03	18400771	93.12	36728
RR5	37935946	93.83	86.53	33533599	88.40	31563842	83.20	23274
RR6	21276042	95.97	93.69	20306633.5	95.44	19920078	93.63	37483
RR7	35570784	94.94	89.04	31696004	89.11	30025680	84.41	27430
RR8	21689934	96.37	94.35	20580676.5	94.89	20153418	92.92	37363
RR9	35093968	95.31	89.64	31801951	90.62	30321993	86.40	26195
RR10	36654365	94.14	87.09	33462054	91.29	31837012	86.86	23714
RR11	21643768	96.26	94.21	20526202	94.84	20149268	93.10	37348
RR12	34935598	95.30	89.67	31878267	91.25	30410198	87.05	25557

**Table 3:** Common nonsynonymous SNPs identified in NDMM and RRMM. The number of NDMM and RRMM patients were listed.

dbSNP	Chromosome	Locus	Ref	Alt	Gene	NDMM	RRMM
rs61821060	chr1	203039046	G	C	PPFIA4	5	11
rs2363468	chr2	208325606	T	C	PIKFYVE	4	11
rs1941635	chr11	118111780	T	G	TMPRSS4	4	10
rs2172521	chr12	57810500	T	C	AVIL	4	10
rs7199961	chr16	88428999	G	C	ZNF469	4	10
rs73714227	chr7	100952147	C	T	MUC3A	4	10
rs4728137	chr7	128815713	C	G	CCDC136	4	10
rs870124	chr1	3411794	T	C	PRDM16	4	9
rs4951168	chr1	205084091	C	T	TMEM81	4	9
rs6491707	chr13	102732665	A	G	CCDC168	4	9
rs35708006	chr15	23441451	T	C	GOLGA6L2	4	9
rs7197779	chr16	10909070	A	G	CIITA	4	9
rs673918	chr17	77194764	A	C	SEC14L1	4	9
rs3746887	chr21	39660813	T	C	B3GALT5	4	9
rs130642	chr22	46281710	T	C	TTC38	4	9
rs9831516	chr3	69180910	G	A	FRMD4B	4	9
rs2261167	chr4	40808730	A	G	NSUN7	4	9
rs4728329	chr7	134541075	A	G	AKR1B10	5	9
rs615474	chr9	35043294	G	T	C9orf131	4	9
rs2215530	chr9	122724689	G	A	OR1L4	4	9
rs3748597	chr1	953279	T	C	NOC2L	4	8
rs10776792	chr1	115033402	A	G	TSHB	4	8
rs1144566	chr1	182600491	T	C	RGS16	4	8
rs28533004	chr1	248650751	T	A	OR2T27	5	8
rs2653588	chr11	8925474	A	G	C11orf16	5	8
rs540687	chr11	57379543	A	G	PRG3	5	8
rs1194099	chr11	65582378	A	T	EHBP1L1	4	8
rs929949	chr12	27696863	A	G	REP15	4	8
rs59122400	chr15	23441526	G	A	GOLGA6L2	5	8
rs8026845	chr15	44674191	T	C	PATL2	4	8
rs8071623	chr17	58543925	G	T	C17orf47	4	8
rs8104843	chr19	15087481	C	G	OR1I1	4	8
rs4806163	chr19	35513204	A	G	DMKN	5	8
rs1059768	chr20	56513348	A	G	RTFDC1	5	8
rs2931761	chr3	112471290	G	T	BTLA	5	8
rs6831040	chr4	81046034	C	T	BMP3	4	8
rs699512	chr7	43771165	G	A	BLVRA	4	8
rs1043708507	chr7	100955225	T	A	MUC3A	4	8
rs3118635	chr9	129098622	G	T	CRAT	4	8
rs10864628	chr1	6575171	A	G	TAS1R1	4	7
rs198400	chr1	11824498	A	G	CLCN6	4	7
rs10480	chr1	150308112	T	C	MRPS21	4	7
rs863363	chr1	158579721	A	G	OR10X1	4	7
rs859398	chr1	175406666	T	C	TNR	4	7
rs2243525	chr1	236543562	G	C	LGALS8	4	7
rs10736251	chr10	116471848	G	A	PNLIPRP3	4	7
rs1897519	chr10	116471851	A	G	PNLIPRP3	4	7
rs7088479	chr10	123746786	T	C	CPXM2	4	7
rs2255246	chr10	133420037	A	G	MTG1	5	7
rs564271	chr11	1835943	T	C	SYT8	4	7
rs10768611	chr11	5151556	A	G	OR52A1	4	7
rs2682123	chr11	6320454	C	G	CAVIN3	4	7
rs2958149	chr12	56716008	A	G	NACA	4	7
rs9300758	chr13	102735870	A	G	CCDC168	4	7
rs9514066	chr13	102875499	G	C	ERCC5	4	7
rs12896533	chr14	19748139	T	C	OR4Q3	4	7
rs1280395	chr15	57439137	A	C	CGNL1	5	7
rs7168069	chr15	68332058	A	C	ITGA11	4	7
rs4787984	chr16	27761580	G	A	KIAA0556	4	7
rs9932770	chr16	29697029	A	G	QPRT	5	7
rs235638	chr16	29780400	G	C	ZG16	4	7
rs4782300	chr16	88431813	C	T	ZNF469	4	7
rs897420	chr17	41514660	G	C	KRT15	4	7
rs2429387	chr17	62689654	G	A	MRC2	4	7
rs1688149	chr17	74866908	C	T	FDXR	4	7
rs820256	chr17	75594749	T	G	MYO15B	4	7
rs2287803	chr19	10001670	T	C	COL5A3	5	7
rs2285422	chr19	36006456	C	G	SYNE4	4	7
rs3103057	chr19	56053798	G	A	NLRP5	4	7
rs2444257	chr2	151465581	A	T	RIF1	4	7
rs6436669	chr2	227248459	A	G	COL4A3	4	7
rs1033545	chr20	18315428	T	A	ZNF133	5	7
rs6076122	chr20	23750857	A	G	CST1	4	7
rs910148	chr20	62881254	T	C	DIDO1	4	7
rs464391	chr21	44579776	G	C	KRTAP10-5	5	7
rs6787916	chr3	52833699	G	C	MUSTN1	4	7
rs28376231	chr5	177503134	G	A	DOK3	4	7
rs28463186	chr7	100995575	A	G	MUC12	4	7
rs6558339	chr8	143249842	T	C	ZFP41	4	7
rs62547039	chr9	34725745	T	C	FAM205A	4	7

## Supplementary Materials

The following are available online at https://www.mdpi.com/2072-6694/11/6/887/s1, Figure S1: Agilent 2100 Bioanalyzer results showed the absence of 18S and 28S rRNA in extracellular RNA (exRNA), Table S1: Differentially Expressed Genese (DEGs) identified in multiple myeloma (MM) patients compared to healthy controls (HC), Table S2: Non-synonymous SNPs identified only in newly diagnosed multiple myeloma (NDMM), Table S3: Non-synonymous SNPs identified only in relapsed and refractory multiple myeloma (RRMM).
